# Numerical Simulation of the Effect of Mg-Doped TiO_2_ Electron Transport Layer on the Performance of Tetragonal MAPbI_3_ Perovskite Solar Cells

**DOI:** 10.3390/nano16191237

**Published:** 2026-09-30

**Authors:** Chunlei Shi, Xuan Yu, Ji Liu, Zhiyuan Chen, Jian Jiao

**Affiliations:** 1 College of Physics, Changchun Normal University, Changchun 130032, China; 2 Changchun Jilin University Zhengyuan Information Technologies Co., Ltd., Changchun 130012, China; ji_liu@live.com

**Keywords:** MAPbI_3_ perovskite solar cells, SCAPS-1D, crystal phase, Mg-doped TiO_2_, charge transport layer, device optimization

## Abstract

A systematic SCAPS-1D simulation was performed to evaluate the effects of charge-transport-layer selection, MAPbI_3_ crystal phase, TiO_2_ modification, and key device parameters on the photovoltaic performance of MAPbI_3_-based perovskite solar cells. Twenty-five combinations of five electron transport layers (ETLs) and five hole transport layers were first screened using tetragonal MAPbI_3_ as the absorber. The TiO_2_/P_3_HT combination exhibited the highest power conversion efficiency (PCE) of 23.653% and was selected for subsequent analysis. Comparison of cubic, orthorhombic, and tetragonal MAPbI_3_ showed that the tetragonal phase delivered the highest PCE, primarily owing to its higher short-circuit current density despite its relatively lower open-circuit voltage. Li-, Co-, Mg-, and Al-doped TiO_2_ ETLs were then evaluated, among which Mg:TiO_2_ produced the largest and most consistent performance improvement, increasing the PCE of the tetragonal device to 23.951%, mainly through an enhancement in fill factor. The selected Glass/FTO/Mg:TiO_2_/tetragonal-MAPbI_3_/P_3_HT/Au device was further optimized by varying the absorber thickness, Mg:TiO_2_ donor concentration, MAPbI_3_ bulk defect density, interface defect density, and back-contact work function. Under the stepwise-selected low-defect conditions, the device achieved a simulated open-circuit voltage of 1.166 V, short-circuit current density of 27.600 mA cm^−2^, fill factor of 87.580%, and PCE of approximately 28.18%. The results highlight the importance of jointly optimizing absorber properties, electron-transport characteristics, defect density, and contact energetics, and they provide a theoretical basis for narrowing the experimental design space of high-performance MAPbI_3_ perovskite solar cells.

## 1. Introduction

Perovskite solar cells (PSCs) have attracted considerable attention in recent years because of their high optical absorption coefficients [1,2,3,4], long carrier diffusion lengths [5,6], tunable band gaps [7], and compatibility with low-temperature solution processing [8,9,10]. Among the various perovskite absorber materials, methylammonium lead iodide (MAPbI_3_) remains one of the most widely investigated due to its suitable direct band gap, strong light-absorption capability [1,2,3,4], and favorable carrier-transport properties [11,12]. However, the photovoltaic performance of MAPbI_3_-based devices is affected by several factors, including the properties of the absorber layer, the selection of charge-transport materials, interfacial carrier recombination, and the electrical characteristics of the electrodes [13]. Optimizing these components is therefore important for improving carrier extraction and overall device performance.

MAPbI_3_ exhibits temperature-dependent structural phase transitions and can exist in orthorhombic, tetragonal, and cubic phases [14,15,16,17,18]. These crystal phases differ in structural and electronic properties, including lattice symmetry, band gap, dielectric constant, and related optoelectronic characteristics, which can influence light absorption and carrier transport [19,20]. Previous studies have demonstrated that structural phase transitions can lead to distinct optical and photovoltaic responses in MAPbI_3_. For example, Zhang et al. investigated MAPbI_3_ solar cells over a temperature range spanning the orthorhombic, tetragonal, and cubic regimes and observed pronounced changes in photocurrent near the orthorhombic transition [21]. Quarti et al. further combined temperature-dependent optical and photovoltaic measurements with first-principles calculations to examine the tetragonal-to-cubic transition and its implications for device behavior [22]. More recently, Gamal et al. [23] directly compared cubic, tetragonal, and orthorhombic MAPbI_3_ using DFT-derived phase-dependent properties coupled with SCAPS-1D simulations in a ZnO/MAPbI_3_/NiOx device architecture. These studies confirm that the crystal phase is an important factor governing MAPbI_3_ photovoltaic behavior. In the present work, the phase comparison is incorporated into a broader device-design procedure, in which the three MAPbI_3_ phases are evaluated within the transport-layer architecture selected from the preceding ETL/HTL screening and the selected phase is subsequently carried forward for TiO_2_ modification and device-parameter optimization.

In addition to the absorber layer, the electron transport layer (ETL) plays an important role in extracting photogenerated electrons and suppressing hole transport toward the front electrode. TiO_2_ is widely used as an ETL in MAPbI_3_-based solar cells because of its suitable energy levels, chemical stability, and electron-transport properties. Nevertheless, its electrical conductivity and interfacial transport characteristics can still limit device performance. More broadly, compositional modification of TiO_2_ has been shown to alter its optoelectronic and interfacial properties. Belkhanchi et al. reported a reduced optical band gap and enhanced electrical conductivity in N-CNT/TiO_2_ composite films, while Ziat et al. further analyzed loading-dependent band alignment in modified TiO_2_/ZnO interfaces using the electron-affinity model [24,25]. Metal-ion modification has therefore been investigated as an effective approach for tuning the electronic and charge-transport properties of TiO_2_. For example, Li-doped TiO_2_ has been reported to exhibit enhanced electrical conductivity and improved electron transport in planar perovskite solar cells [26], while Li- and Co-treated TiO_2_ have also been evaluated in MAPbI_3_-based devices using SCAPS-1D simulations [27]. Al- and Mg-doped mesoporous TiO_2_ have been experimentally investigated in CH_3_NH_3_PbI_3_ solar cells [28], and their effects as modified ETLs in MAPbI_3_ devices have subsequently been analyzed numerically [29]. These studies indicate that the photovoltaic response to TiO_2_ modification is dopant-dependent because different dopants alter the electronic and transport parameters of the ETL to different extents. A direct comparison of Li-, Co-, Mg-, and Al-modified TiO_2_ under the same simulation framework is therefore useful for evaluating their relative effects under consistent device conditions. In the present model, these four modified TiO_2_ layers are compared using the same MAPbI_3_-based device framework.

Although charge-transport-layer selection, MAPbI_3_ crystal phase, TiO_2_ modification, and individual device parameters have been investigated in previous studies, these factors are generally examined separately. Consequently, their relative contributions to device performance and the dominant limitations at different stages of device optimization remain difficult to compare directly. A unified simulation framework therefore provides an opportunity not only to identify a favorable device configuration, but also to distinguish the roles of absorber properties, charge-transport characteristics, recombination losses, and contact energetics in determining overall photovoltaic performance. In this work, SCAPS-1D is employed to investigate planar Glass/FTO/ETL/MAPbI_3_/HTL/Au perovskite solar cells. First, five electron transport layers and five hole transport layers are combined to construct 25 device configurations, and the most suitable ETL/HTL combination is selected using tetragonal MAPbI_3_ as the absorber. The photovoltaic performance of orthorhombic, tetragonal, and cubic MAPbI_3_ is then compared. Subsequently, Li-, Co-, Mg-, and Al-modified TiO_2_ layers are evaluated to identify a suitable TiO_2_ modification strategy. Finally, the selected device is further optimized by varying the MAPbI_3_ absorber thickness, the donor concentration of the modified TiO_2_ layer, the MAPbI_3_ bulk defect density, the MAPbI_3_/ETL interface defect density, and the back-contact work function. Through this stepwise numerical analysis, the effects of material selection and key device parameters on the photovoltaic performance of MAPbI_3_-based solar cells are systematically evaluated. The results are expected to provide useful theoretical guidance for the selection of charge-transport materials and the optimization of key device parameters, thereby helping to narrow the experimental design space for high-performance MAPbI_3_ perovskite solar cells.

## 2. Materials and Methods

### 2.1. Device Architecture

A planar MAPbI_3_ perovskite solar-cell model with a Glass/FTO/TiO_2_/MAPbI_3_/P_3_HT/Au configuration was established using SCAPS-1D simulation software (version 3.3.10), as illustrated in Figure S1. Illumination was introduced from the Glass/FTO side. The glass layer served as the transparent substrate, while FTO acted as the transparent front contact for light transmission and electron collection. TiO_2_ was employed as the ETL to extract photogenerated electrons from the MAPbI_3_ absorber and suppress hole transport toward the front contact. MAPbI_3_ served as the photoactive absorber layer, whereas P_3_HT was used as the hole transport layer (HTL) for selective hole extraction. Au was employed as the back contact.

Upon illumination, photon absorption in the MAPbI_3_ layer generates electron–hole pairs. The photogenerated carriers are subsequently separated and transported through the corresponding charge-selective layers, with electrons transferred through the ETL toward the FTO contact and holes transported through the HTL toward the Au back contact. This device architecture was used as the basic configuration for the subsequent screening and optimization simulations.

### 2.2. Numerical Simulation Framework

SCAPS-1D is a one-dimensional numerical simulation program developed for the analysis of thin-film photovoltaic devices. It calculates the electrostatic potential, carrier concentrations, current densities, energy-band distributions, recombination behavior, and current density–voltage (J–V) characteristics by self-consistently solving Poisson’s equation together with the electron and hole continuity equations [30]. Poisson’s equation is expressed as:

(1) $$\frac{d}{dx}\left(\varepsilon \frac{d\varphi }{dx}\right)=-q(p-n+N_{D}^{+}-N_{A}^{-}+\rho_{def})$$

where φ is the electrostatic potential, *q* is the elementary charge, ε is the dielectric permittivity, *n* and *p* are the electron and hole concentrations, respectively, $N_{D}^{+}$ and $N_{A}^{-}$ denote the ionized donor and acceptor concentrations, and $\rho_{def}$ represents the charge density associated with defects [31]. The electron and hole continuity equations are given by:

(2) $$\frac{{dJ}_{n}}{dx}=q(G-R)$$

(3) $$\frac{{dJ}_{p}}{dx}=-q(G-R)$$

where *J_n_* and *J_p_* are the electron and hole current densities, respectively, *G* is the photogeneration rate, and *R* is the carrier recombination rate. Carrier transport is described by the combined contributions of drift and diffusion:

(4) $$J_{n}=q\mu_{n}nE+qD_{n}\frac{dn}{dx}$$

(5) $$J_{p}=q\mu_{p}pE-qD_{p}\frac{dp}{dx}$$

where $\mu_{n}$ and $\mu_{p}$ represent the electron and hole mobilities, respectively; *D_n_* and *D_p_* are the corresponding diffusion coefficients, and *E* is the internal electric field.

All simulations were performed under standard AM1.5G illumination with an incident power density of 1000 W m^−2^ at an operating temperature of 300 K. Unless otherwise specified, all material, interface, and contact parameters other than the parameter being investigated were kept unchanged within each comparative simulation to ensure consistency among the calculated devices.

### 2.3. Material and Interface Parameters

The material parameters used in the SCAPS-1D simulations were obtained from previously reported studies and are summarized in Tables S1–S5. These parameters include layer thickness, band gap (E_g_), electron affinity (χ), relative dielectric permittivity (ε_r_), effective density of states in the conduction and valence bands (N_C_ and N_V_), electron and hole thermal velocities, carrier mobilities, shallow donor and acceptor concentrations, and bulk defect density.

Five candidate HTLs, namely P_3_HT, Zn_3_P_2_, CBTS, CuSbS_2_, and GO, were considered for the transport-layer screening. Their input parameters are summarized in Table S1 [26]. The electronic properties of these materials, particularly their band-gap energies, electron affinities, carrier mobilities, and acceptor concentrations, determine their energy-level alignment with MAPbI_3_ and their ability to selectively transport holes.

Similarly, TiO_2_, SnS_2_, WS_2_, C_60_, and MoO_3_ were evaluated as candidate ETLs. Their material parameters are listed in Table S2. Differences in electron affinity and band gap affect the energetic alignment between the ETL and MAPbI_3_, whereas carrier mobility, doping concentration, and defect density influence charge transport and recombination within the transport layer. The parameters listed in Tables S1 and S2 were maintained during the initial screening of the 25 ETL/HTL combinations.

Defect states were introduced at the MAPbI_3_/HTL and ETL/MAPbI_3_ interfaces to account for interfacial carrier trapping and recombination. The interface-defect parameters included defect type, energetic distribution, defect energy level, defect density, and electron and hole capture cross sections, as summarized in Table S3. During the initial transport-layer screening and crystal-phase comparison, the interface parameters were kept fixed. The defect density at the MAPbI_3_/ETL interface was varied only in the subsequent interface-defect analysis.

To evaluate the influence of the MAPbI_3_ crystal phase, cubic, orthorhombic, and tetragonal MAPbI_3_ were introduced into the same device architecture. The absorber thickness was fixed at 0.60 μm for all three phases to ensure a consistent comparison, while the phase-dependent material parameters were adopted from the literature and are summarized in Table S4 [23,24,27]. The parameters used for Co-, Al-, Li-, and Mg-modified TiO_2_ are listed in Table S5 [27]. These parameter sets were subsequently employed to compare the photovoltaic responses associated with different MAPbI_3_ phases and TiO_2_ modification strategies.

### 2.4. Simulation Procedure

A stepwise simulation procedure was adopted to evaluate the effects of material selection and key device parameters on photovoltaic performance. Tetragonal MAPbI_3_ was initially used as the reference absorber. Five ETLs (TiO_2_, SnS_2_, WS_2_, C_60_, and MoO_3_) and five HTLs (P_3_HT, Zn_3_P_2_, CBTS, CuSbS_2_, and GO) were combined to construct 25 ETL/HTL configurations. Their photovoltaic performances were evaluated in terms of the open-circuit voltage (V_OC_), short-circuit current density (J_SC_), fill factor (FF), and power conversion efficiency (PCE). The transport-layer combination showing the best overall photovoltaic performance was selected as the reference configuration for the subsequent simulations.

The selected ETL/HTL architecture was subsequently applied to devices based on cubic, orthorhombic, and tetragonal MAPbI_3_. To isolate the effect of the absorber crystal phase, the MAPbI_3_ thickness was fixed at 0.60 μm, and the transport-layer, interface, contact, illumination, and operating-temperature conditions were kept unchanged. Only the phase-dependent absorber parameters listed in Table S4 were varied. The resulting V_OC_, J_SC_, FF, and PCE values were compared to evaluate the influence of MAPbI_3_ crystal phase on device performance.

After selection of the MAPbI_3_ phase, TiO_2_ layers modified with Li, Co, Mg, and Al were introduced individually into the device model using the material parameters given in Table S5. Their photovoltaic responses were compared with those of the corresponding unmodified TiO_2_ device. The most favorable TiO_2_ modification was subsequently selected for further device optimization.

Finally, the selected device configuration was systematically optimized by varying the MAPbI_3_ absorber thickness, the doping concentration of the modified TiO_2_ layer, the MAPbI_3_ bulk defect density, the MAPbI_3_/ETL interface defect density, and the back-contact work function. During each parameter scan, the parameter under investigation was varied within the specified range while the remaining simulation parameters were held constant. The resulting changes in V_OC_, J_SC_, FF, and PCE were analyzed to determine suitable parameter ranges and establish the optimized device configuration.

## 3. Results and Discussion

### 3.1. Screening and Energy-Band Analysis of ETL/HTL Combinations

**Figure 1:**
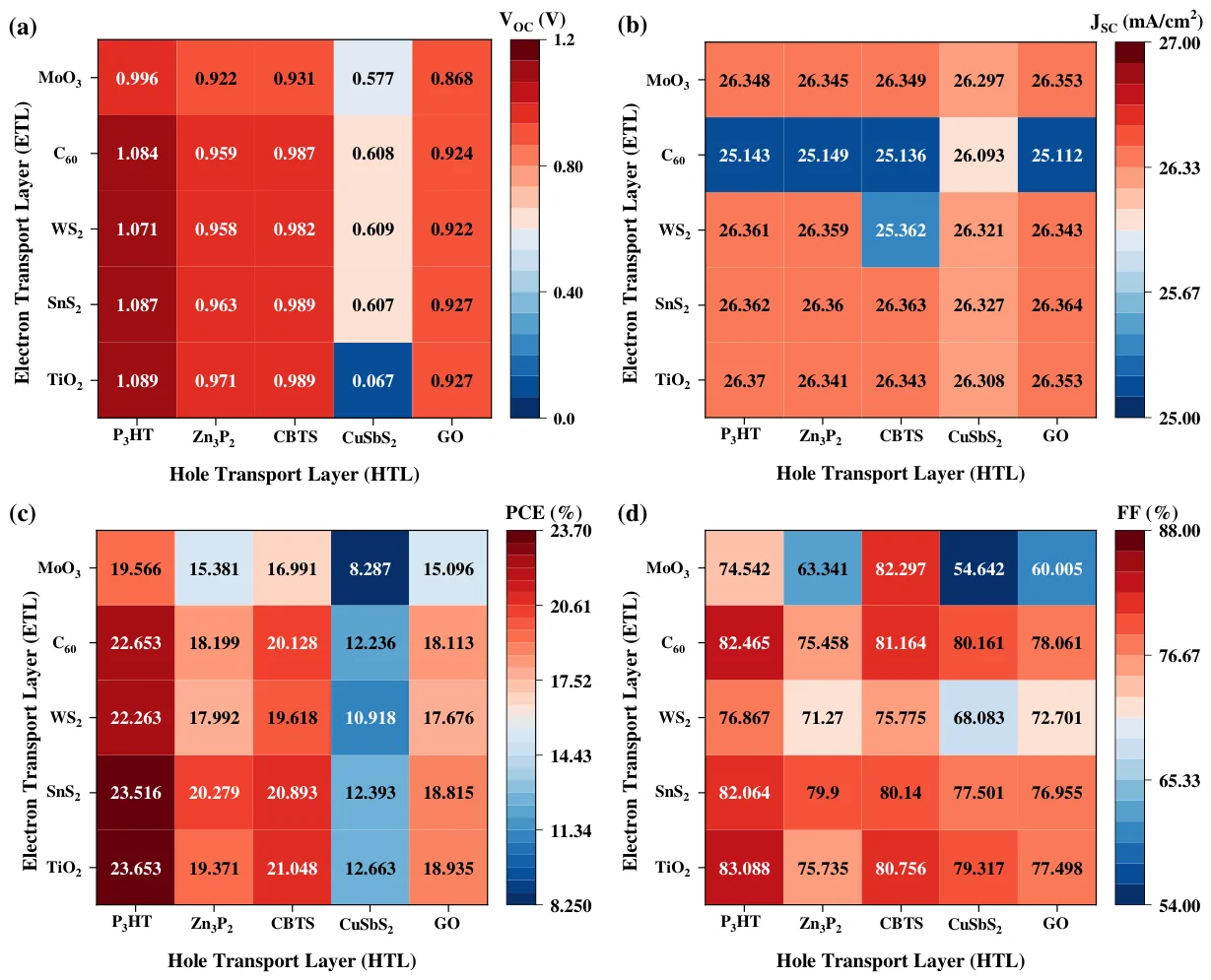
Photovoltaic performance of tetragonal MAPbI_3_-based solar cells with different ETL/HTL combinations: (**a**) V_OC_, (**b**) J_SC_, (**c**) PCE, and (**d**) FF.

To identify a suitable charge-transport-layer configuration for tetragonal MAPbI_3_-based solar cells, five electron transport layers (TiO_2_, SnS_2_, WS_2_, C_60_, and MoO_3_) and five hole transport layers (P_3_HT, Zn_3_P_2_, CBTS, CuSbS_2_, and GO) were combined to construct 25 ETL/HTL configurations. During the screening process, the absorber properties, layer thicknesses, interface parameters, contact conditions, illumination intensity, and operating temperature were kept unchanged, while only the ETL and HTL materials were varied. The resulting V_OC_, J_SC_, PCE, and FF values are summarized in Figure 1.

#### 3.1.1. Photovoltaic Performance of Different ETL/HTL Combinations

As shown in Figure 1, the photovoltaic parameters vary considerably among the 25 ETL/HTL combinations, demonstrating that the selection of charge-transport materials has a pronounced influence on the simulated device performance. In particular, the variation in PCE does not follow the change in any single transport-layer parameter, indicating that the overall photovoltaic response results from the combined effects of energy-level alignment, carrier-selective transport, and recombination losses at the transport-layer/absorber interfaces. Among the investigated configurations, the TiO_2_/P_3_HT combination yields the highest PCE of 23.653%. P_3_HT-based devices generally exhibit favorable efficiencies when combined with several of the investigated ETLs, while the highest overall performance is obtained when TiO_2_ is used as the electron-selective contact. This result suggests that the TiO_2_/MAPbI_3_ and MAPbI_3_/P_3_HT interfaces provide a suitable combination of electron and hole selectivity under the adopted simulation parameters.

From an energetic perspective, TiO_2_ provides an electron-selective contact with MAPbI_3_, whereas its relatively deep valence-band position restricts hole transport toward the front contact. On the opposite side of the absorber, the energy levels of P_3_HT favor hole extraction while providing a barrier against electron transfer into the HTL. The complementary selectivity of the two transport layers is therefore consistent with the superior photovoltaic performance obtained for the TiO_2_/MAPbI_3_/P_3_HT configuration [32]. It should be emphasized, however, that the calculated PCE reflects the combined influence of all material and interface parameters used in the model rather than energy-level alignment alone. Based on the transport-layer screening, the Glass/FTO/TiO_2_/MAPbI_3_/P_3_HT/Au configuration was selected as the reference device for the subsequent comparison of MAPbI_3_ crystal phases and TiO_2_ modification strategies.

#### 3.1.2. Energy-Band Alignment of the Selected TiO_2_/MAPbI_3_/P_3_HT Device

**Figure 2:**
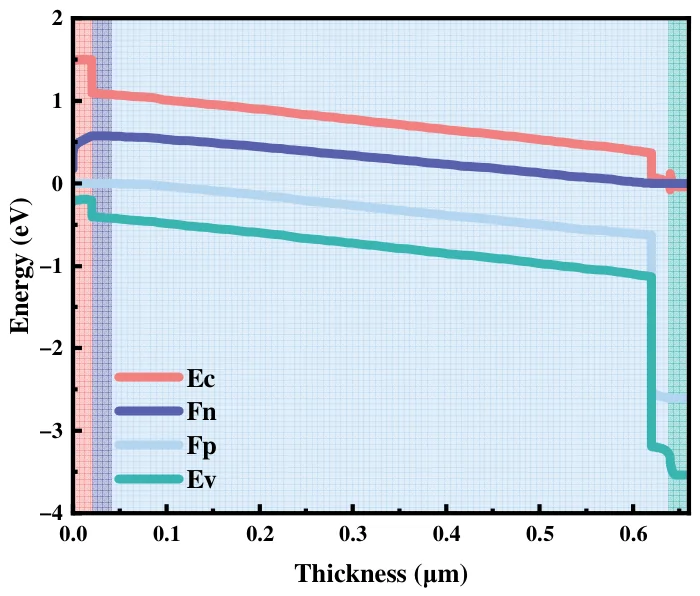
Simulated energy-band diagram of the Glass/FTO/TiO_2_/tetragonal-MAPbI_3_/P_3_HT/Au device under illumination, showing the conduction-band minimum (EC), valence-band maximum (EV), electron quasi-Fermi level (E_Fn_), and hole quasi-Fermi level (E_Fp_).

To further examine the carrier-selective characteristics of the selected transport-layer configuration, the simulated energy-band distribution of the Glass/FTO/TiO_2_/tetragonal-MAPbI_3_/P_3_HT/Au device under illumination is shown in Figure 2. The conduction-band minimum (E_C_) and valence-band maximum (E_V_), together with the electron and hole quasi-Fermi levels (E_Fn_ and E_Fp_), are presented across the device. The separation between E_Fn_ and E_Fp_ under illumination reflects the nonequilibrium carrier population generated within the photovoltaic device.

The relative band-edge positions can be estimated from the electron affinity (χ) and band gap (Eg), according to E_C_ = −χ and E_V_ = −(χ + Eg). In this work, the conduction-band offset and valence-band offset are defined as ΔE_C_ = E_C_(transport layer) − E_C_(MAPbI_3_) and ΔE_V_ = E_V_(transport layer) − E_V_(MAPbI_3_). A positive ΔE_C_ indicates that the transport layer conduction band lies above that of MAPbI_3_, forming an electron-blocking barrier, while a negative ΔE_C_ favors electron extraction. A positive ΔE_V_ favors hole transfer, while a large negative ΔE_V_ indicates a hole-blocking barrier. At the P_3_HT/MAPbI_3_ interface, the conduction-band offset is ΔE_C_ = +0.40 eV, indicating that the P3HT conduction band lies 0.40 eV above that of tetragonal MAPbI_3_. This positive conduction-band offset forms an energetic barrier against electron transfer from MAPbI_3_ into the P_3_HT layer, thereby supporting the electron-blocking function of the HTL. The valence-band offset is ΔE_V_ = +0.20 eV, indicating that the P_3_HT valence band lies 0.20 eV above that of MAPbI_3_, which is favorable for hole transfer from the absorber to P_3_HT.

At the MAPbI_3_/TiO_2_ interface, the conduction-band offset is ΔE_C_ = −0.10 eV, indicating that the TiO_2_ conduction band lies 0.10 eV below that of MAPbI_3_. This negative offset provides an energetically favorable direction for electron transfer from the absorber to the ETL. The valence-band offset is ΔE_V_ = −1.80 eV, indicating that the TiO_2_ valence band lies 1.80 eV below that of MAPbI_3_. This large negative offset introduces a substantial energetic barrier for holes, thereby restricting their transfer into the electron transport layer. TiO_2_ therefore acts as an electron-selective contact on the front side of the absorber. The magnitude of the conduction-band offset at this interface is also relevant to interfacial carrier accumulation and recombination, particularly when the interface defect density is high; this effect is examined further in the subsequent interface-defect analysis.

Within the MAPbI_3_ absorber, both the conduction and valence bands exhibit continuous bending while remaining approximately parallel, indicating that the band gap is essentially maintained across the absorber and that the variation in band-edge position mainly originates from the internal electrostatic potential. Such band bending is consistent with the built-in electric field established across the device, which assists the directional transport of electrons toward TiO_2_ and holes toward P_3_HT. Because tetragonal MAPbI_3_ is modeled as a weakly p-type absorber with a relatively low acceptor concentration N_A_ = 1.0 × 10^11^ cm^−3^, its internal band profile is strongly influenced by the electrostatic conditions imposed by the two selective contacts.

Under illumination, clear splitting of the electron and hole quasi-Fermi levels is observed within the device. The electron quasi-Fermi level remains closer to the conduction band, whereas the hole quasi-Fermi level is located closer to the valence band. The magnitude of this quasi-Fermi-level splitting is associated with the photovoltage generated by the device and is sensitive to carrier recombination. Together with the band-edge alignment, these features indicate that the TiO_2_/MAPbI_3_/P_3_HT architecture provides complementary electron- and hole-selective contacts: electrons are preferentially extracted through TiO_2_ toward FTO, whereas holes are transported through P_3_HT toward the back contact. Overall, the transport-layer screening and energy-band analysis support the selection of TiO_2_/P_3_HT as the reference ETL/HTL combination for tetragonal MAPbI_3_. The resulting PCE of 23.653% arises from the combined effects of favorable charge-selective contacts, carrier transport, and the interface properties adopted in the numerical model. This configuration was therefore used as the basis for the subsequent investigation of MAPbI_3_ crystal-phase effects and TiO_2_ modification.

### 3.2. Effect of MAPbI_3_ Crystal Phase on Photovoltaic Performance

To evaluate the influence of the MAPbI_3_ crystal phase on device performance, cubic, orthorhombic, and tetragonal MAPbI_3_ were employed as the absorber layer in the selected Glass/FTO/TiO_2_/MAPbI_3_/P_3_HT/Au architecture. The absorber thickness was fixed at 0.60 μm for all three phases, while the transport-layer, interface, contact, illumination, and operating-temperature conditions were kept unchanged. The calculated photovoltaic parameters are summarized in Table 1.

The three MAPbI_3_ phases exhibit distinct photovoltaic responses. The tetragonal device achieves the highest PCE of 23.653%, followed by the orthorhombic (21.599%) and cubic (19.484%) devices. Notably, the higher efficiency of the tetragonal device does not originate from simultaneous improvement in all photovoltaic parameters. In fact, its V_OC_ (1.089 V) and FF (82.465%) are slightly lower than those of the orthorhombic and cubic devices. Its superior PCE is primarily associated with the substantially higher J_SC_ of 26.343 mA cm^−2^, compared with 22.473 mA cm^−2^ for the orthorhombic phase and 20.221 mA cm^−2^ for the cubic phase.

This variation in J_SC_ is consistent with the different band gaps adopted for the three MAPbI_3_ phases. The tetragonal, orthorhombic, and cubic absorbers have band gaps of 1.50, 1.62, and 1.70 eV, respectively. Under otherwise comparable device conditions, the smaller band gap of tetragonal MAPbI_3_ allows photons over a broader energy range to contribute to photocarrier generation, resulting in a higher simulated photocurrent. As the band gap increases from the tetragonal to the orthorhombic and cubic phases, the calculated J_SC_ decreases accordingly. This trend is consistent with the progressively reduced contribution of lower-energy photons to carrier generation.

The V_OC_ trend, however, is opposite to that of J_SC_. The cubic and orthorhombic devices exhibit higher V_OC_ values than the tetragonal device, which is consistent with the general dependence of the achievable photovoltage on absorber band gap and recombination losses. Thus, the phase-dependent device performance reflects a balance between photocurrent generation and voltage output. In the present simulations, the gain in J_SC_ provided by tetragonal MAPbI_3_ is sufficiently large to compensate for its lower V_OC_ and slightly reduced FF, leading to the highest overall PCE.

It is also important to note that, according to the adopted material parameter sets, the three MAPbI_3_ phases share the same electron affinity, carrier mobilities, effective densities of states, and bulk defect density, whereas their band gaps and dielectric permittivities differ. Therefore, within the assumptions of the present SCAPS-1D model, the observed differences in photovoltaic performance are mainly governed by the phase-dependent parameters included in the model, particularly the band-gap variation [24]. This simplified parameterization does not capture the full range of phase-dependent mobilities, defect densities, electron affinities, and other electronic properties reported in the literature. Accordingly, the calculated phase ranking is specific to the adopted parameter sets and should not be interpreted as a universal intrinsic ranking of MAPbI_3_ phases. Under these model assumptions, tetragonal MAPbI_3_ gives the highest simulated PCE of 23.653% and is therefore selected for the subsequent analysis.

### 3.3. Effect of Metal-Doped TiO_2_ ETLs on Device Performance

Based on the selected TiO_2_/P_3_HT transport-layer architecture, the effects of Li-, Co-, Mg-, and Al-doped TiO_2_ ETLs were further evaluated for devices employing cubic, orthorhombic, and tetragonal MAPbI_3_ absorbers. For each crystal phase, the corresponding undoped TiO_2_ device was used as the reference, while the absorber, HTL, interface, contact, and illumination parameters were kept unchanged. The resulting photovoltaic parameters were compared to identify a suitable TiO_2_ modification strategy for subsequent device optimization.

#### 3.3.1. Photovoltaic Response to Different TiO_2_ Dopants

The photovoltaic parameters obtained using Li- and Co-doped TiO_2_ are summarized in Table 2. Both modifications result in only moderate changes in V_OC_ and J_SC_, whereas a more noticeable increase is observed in FF. For tetragonal MAPbI_3_, for example, Li:TiO_2_ increases the FF from 82.465% to 83.092% and the PCE from 23.653% to 23.768%, while Co: TiO_2_ gives an FF of 83.055% and a PCE of 23.760%. Similar trends are observed for the orthorhombic and cubic devices.

The relatively limited improvements obtained with Li- and Co-doped TiO_2_ can be understood in relation to the electronic parameters adopted for these ETLs. Both modified TiO_2_ layers have a band gap of 3.40 eV and an electron affinity of 3.90 eV, while their electron mobilities are substantially lower than those used for Mg: TiO_2_. In particular, the electron mobility of Li: TiO_2_ is only 0.02 cm^2^ V^−1^ s^−1^, and that of Co: TiO_2_ is 3.3 cm^2^ V^−1^ s^−1^. Such relatively low electron mobilities can limit charge transport through the ETL and thereby offset part of the potential benefit arising from the altered electronic structure. The simulated results are consistent with this interpretation: neither Li nor Co substantially increases J_SC_, and their overall efficiency gains remain modest.

Mg-doped TiO_2_ produces the largest improvement among the four investigated modifications. As shown in Table 2, the PCE increases from 19.484% to 19.834% for cubic MAPbI_3_, from 21.599% to 21.919% for orthorhombic MAPbI_3_, and from 23.653% to 23.951% for tetragonal MAPbI_3_. In contrast to the relatively small variations in V_OC_ and J_SC_, the FF increases appreciably for all three phases. For example, the FF of the tetragonal device increases from 82.465% to 83.612%, while V_OC_ and J_SC_ change only from 1.089 to 1.087 V and from 26.343 to 26.341 mA cm^−2^, respectively.

The SCAPS results show that Mg modification mainly improves the FF, while V_OC_ and J_SC_ remain nearly unchanged. These changes in the photovoltaic parameters are direct outputs of the numerical simulation. According to the adopted material parameters, Mg:TiO_2_ combines a high donor concentration (N_D_ = 9 × 10^20^ cm^−3^) with an electron mobility of 20 cm^2^ V^−1^ s^−1^. The attribution of the FF improvement to enhanced ETL conductivity, reduced resistive transport losses, and more efficient electron transport is therefore a physical interpretation based on these input parameters rather than a direct microscopic result of SCAPS. It should also be noted that the donor concentration and electron mobility adopted for Mg:TiO_2_ are literature-derived input parameters rather than quantities obtained from a microscopic Mg-doping model. Their values can depend strongly on Mg concentration and preparation conditions, and the present simulation does not establish that N_D_ = 9 × 10^20^ cm^−3^ and μe = 20 cm^2^ V^−1^ s^−1^ can necessarily be achieved simultaneously in a specific experimental film. Therefore, the simulated advantage of Mg:TiO_2_ should be interpreted as arising from the adopted transport-parameter combination rather than from an intrinsic superiority of Mg doping itself [33].

In comparison, Al-doped TiO_2_ produces only negligible changes in photovoltaic performance. As shown in Table 2, the PCE values of the cubic, orthorhombic, and tetragonal devices are 19.490%, 21.607%, and 23.660%, respectively, differing from the corresponding undoped devices by less than 0.01 percentage points. The V_OC_ and J_SC_ values are essentially unchanged, while only marginal variations in FF are observed. These results indicate that, under the adopted simulation conditions, the electronic parameters of Al:TiO_2_ do not provide a substantial additional benefit to carrier transport or collection compared with the undoped reference. Overall, the comparison of the four TiO_2_ modifications shows that their influence is manifested primarily through changes in the FF rather than through pronounced variations in V_OC_ or J_SC_. Among the investigated literature-derived parameter sets, Mg:TiO_2_ provides the highest simulated performance, mainly because of its favorable combination of donor concentration and electron mobility [34,35].

#### 3.3.2. Comparison of TiO_2_ Doping Effects Across MAPbI3 Crystal Phases

**Figure 3:**
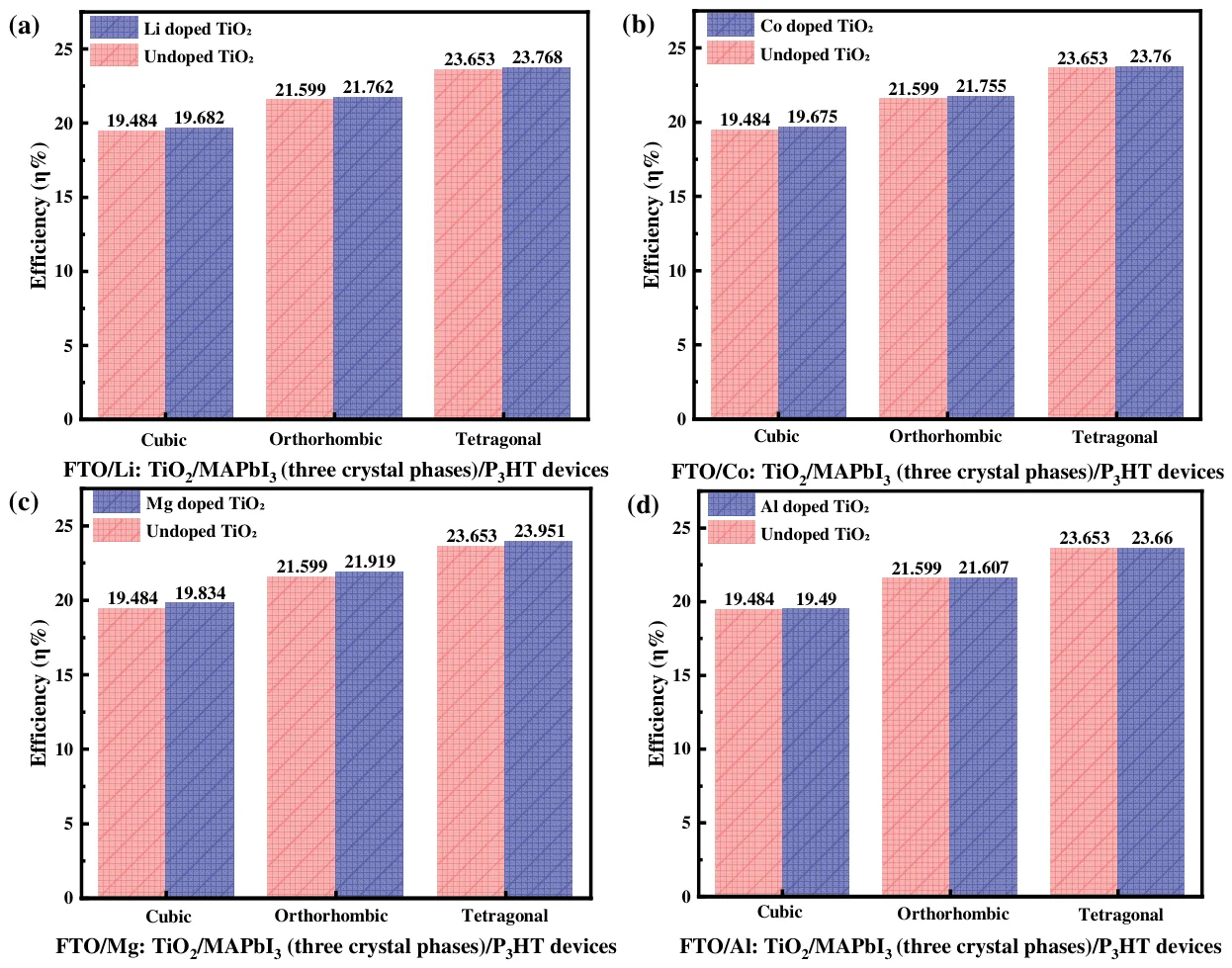
Comparison of the PCE of MAPbI_3_-based devices employing undoped and metal-doped TiO_2_ ETLs for different absorber crystal phases: (**a**) Li:TiO_2_, (**b**) Co:TiO_2_, (**c**) Mg:TiO_2_, and (**d**) Al:TiO_2_.

Figure 3 compares the PCE values of devices employing undoped and metal-doped TiO_2_ for the three MAPbI_3_ crystal phases. Although all four TiO_2_ modifications produce different degrees of performance change, the original phase-dependent efficiency ranking remains unchanged: tetragonal MAPbI_3_ consistently exhibits the highest PCE, followed by the orthorhombic and cubic phases. This indicates that TiO_2_ modification improves the transport-layer characteristics without altering the overall performance trend established by the absorber crystal phase. Among the four dopants, Mg provides the largest PCE enhancement for all three phases. The PCE increases by 0.298 percentage points for tetragonal MAPbI_3_, 0.320 percentage points for the orthorhombic phase, and 0.350 percentage points for the cubic phase. These correspond to relative improvements of approximately 1.26%, 1.48%, and 1.80%, respectively.

Li and Co also produce positive but smaller efficiency gains. For tetragonal MAPbI_3_, Li and Co increase the PCE by approximately 0.115 and 0.107 percentage points, respectively. The corresponding improvements are somewhat larger for the cubic and orthorhombic phases but remain below those obtained with Mg modification. Al doping has the weakest influence, with PCE changes of only 0.006–0.008 percentage points across the three phases. Thus, the effect of ETL modification depends strongly on the electronic parameters associated with the selected dopant, with Mg:TiO_2_ providing the most favorable overall response under the present simulation conditions.

It is worth noting that the largest relative improvement induced by Mg doping occurs for the cubic device (approximately 1.80%), whereas the tetragonal device shows the smallest relative enhancement (approximately 1.26%). This does not imply that Mg is intrinsically more effective for cubic MAPbI_3_; rather, the larger percentage increase partly reflects the lower baseline efficiency of the cubic device. In terms of absolute device performance, the Mg:TiO_2_/tetragonal-MAPbI_3_/P_3_HT configuration remains clearly superior, reaching a PCE of 23.951%, compared with 21.919% and 19.834% for the corresponding orthorhombic and cubic devices.

Taken together, the results show that the absorber crystal phase and TiO_2_ modification play different roles in device performance. The MAPbI_3_ phase largely determines the baseline photovoltaic output, particularly through its influence on JSC, whereas TiO_2_ modification provides a smaller secondary optimization that is mainly reflected in FF. Importantly, none of the four TiO_2_ modifications changes the phase-dependent performance ranking. These results suggest a sequential design criterion: absorber selection should first establish a favorable photocurrent baseline, after which ETL modification should focus on improving transport-related parameters such as carrier concentration and mobility. Based on this criterion, Mg:TiO_2_ was selected for subsequent optimization because it provided the highest PCE among the four investigated parameter sets.

### 3.4. Optimization of the Mg:TiO_2_/Tetragonal-MAPbI_3_ Device

Following the transport-layer, crystal-phase, and TiO_2_-modification screening described above, the Glass/FTO/Mg:TiO_2_/tetragonal-MAPbI_3_/P_3_HT/Au configuration was selected for further optimization. The effects of absorber thickness, Mg:TiO_2_ donor concentration, MAPbI_3_ bulk defect density, MAPbI_3_/Mg:TiO_2_ interface defect density, and back-contact work function were investigated sequentially. Unless otherwise specified, only the parameter under investigation was varied in each simulation step, while the remaining parameters were kept unchanged. This one-at-a-time approach was intended to identify the individual influence and suitable range of each parameter rather than to determine a global optimum in the full multidimensional parameter space. Possible coupling among these parameters is therefore not explicitly considered in the present model.

#### 3.4.1. Energy-Band Characteristics of the Mg:TiO_2_/Tetragonal-MAPbI_3_ Device

**Figure 4:**
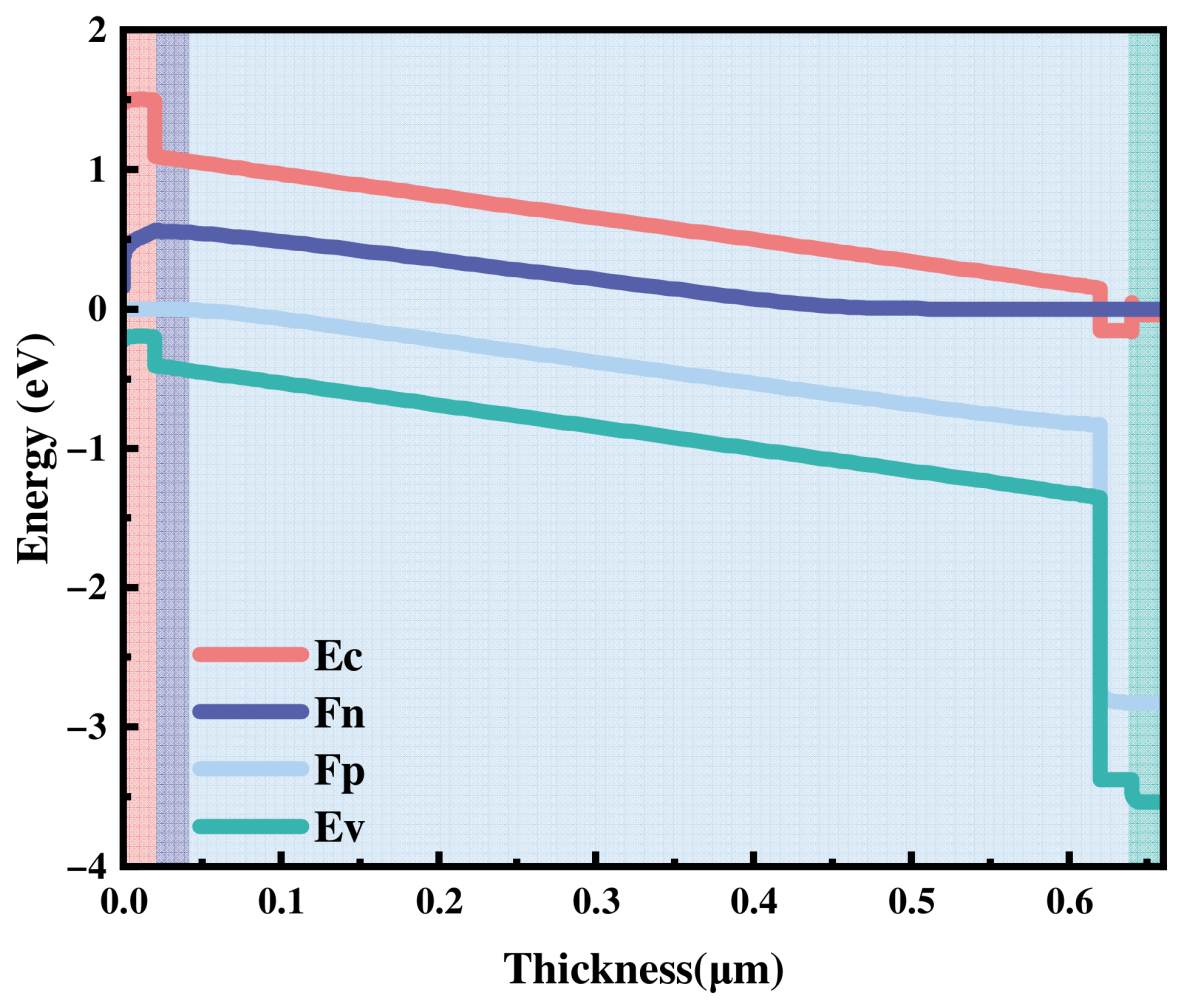
Simulated energy-band diagram of the Glass/FTO/Mg:TiO_2_/tetragonal-MAPbI_3_/P3HT/Au device under illumination.

Figure 4 shows the simulated energy-band distribution of the selected Mg:TiO_2_/tetragonal-MAPbI_3_/P_3_HT device under illumination. According to the adopted material parameters, Mg:TiO_2_ has a band gap of 3.22 eV and an electron affinity of 4.20 eV. Combined with the electron affinity of tetragonal MAPbI_3_, this results in a conduction-band offset of approximately ΔE_C_= −0.30 eV at the MAPbI_3_/Mg:TiO_2_ interface. The lower conduction-band position of Mg:TiO_2_ provides an energetically favorable pathway for electron transfer from the absorber toward the ETL, while the large valence-band offset suppresses hole transfer into Mg:TiO_2_. The interface therefore maintains the electron-selective and hole-blocking characteristics required for efficient carrier extraction.

A notable feature of the adopted Mg:TiO_2_ parameter set is its high donor concentration and electron mobility. SCAPS directly predicts the resulting device-level photovoltaic response and energy-band profile, but it does not model the microscopic origin of these material parameters. Therefore, the interpretation that the high donor concentration and mobility improve ETL conductivity and electron transport should be regarded as a physical explanation consistent with the simulated increase in FF, rather than as a direct microscopic result of the SCAPS calculation [36].

The MAPbI_3_ absorber retains a continuously bent band profile under illumination, while a clear separation between the electron and hole quasi-Fermi levels is observed. Together with the complementary band alignment provided by Mg:TiO_2_ and P_3_HT, this energy-band configuration supports selective transport of electrons toward the front contact and holes toward the back contact. The selected Mg:TiO_2_/tetragonal-MAPbI_3_/P_3_HT architecture was therefore used for the subsequent parameter optimization.

#### 3.4.2. Effect of MAPbI_3_ Absorber Thickness

**Figure 5:**
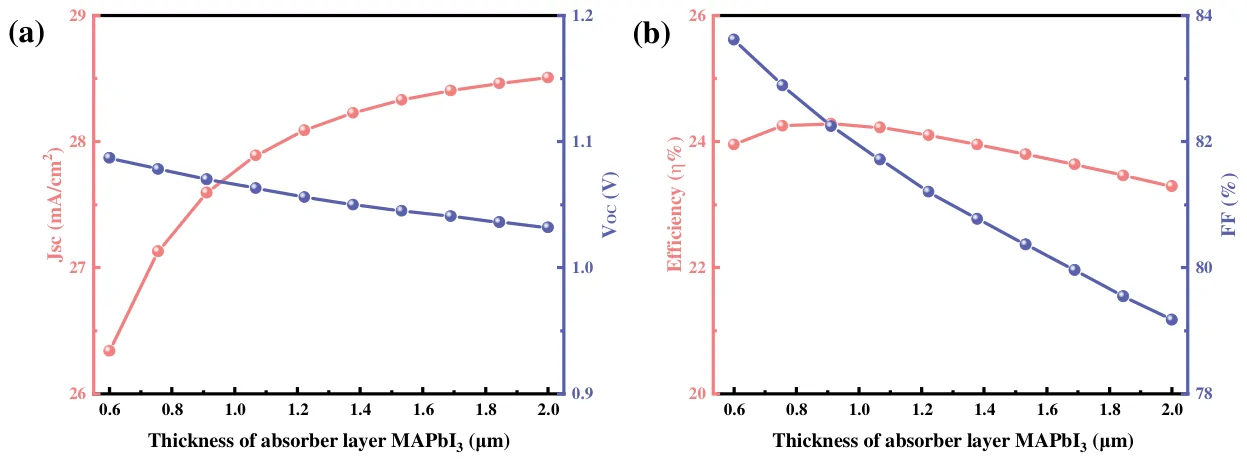
Effect of MAPbI_3_ absorber thickness on (**a**) V_OC_ and J_SC_ and (**b**) FF and PCE.

The absorber thickness directly affects both photon absorption and carrier-transport distance and is therefore an important parameter for balancing photocarrier generation and recombination losses. To determine a suitable MAPbI_3_ thickness, the absorber layer was varied from 0.60 to 2.00 μm while the remaining device parameters were kept unchanged. The corresponding changes in V_OC_, J_SC_, FF, and PCE are shown in Figure 5. As the MAPbI_3_ thickness increases, J_SC_ increases progressively, which is consistent with enhanced absorption of incident photons in a thicker absorber layer. In contrast, V_OC_ and FF gradually decrease with increasing thickness [37]. A thicker absorber increases the transport distance that photogenerated carriers must travel before reaching the selective contacts and increases the probability of bulk recombination, thereby offsetting part of the photocurrent gain [38].

As a result of these competing effects, the PCE exhibits a non-monotonic dependence on absorber thickness. At relatively low thicknesses, the increase in J_SC_ dominates the photovoltaic response, resulting in an improvement in PCE. As the absorber becomes thicker, the incremental gain in photogeneration diminishes, whereas carrier-transport and recombination losses continue to increase. The PCE therefore reaches a maximum before decreasing at larger thicknesses. Within the investigated range, the maximum PCE of 24.282% is obtained at an absorber thickness of 0.911 μm, corresponding to a V_OC_ of 1.070 V, a J_SC_ of approximately 27.594 mA cm^−2^, and an FF of 82.241%. Accordingly, 0.911 μm was adopted as the optimized absorber thickness for the subsequent simulations.

#### 3.4.3. Effect of Mg:TiO_2_ Donor Concentration

**Figure 6:**
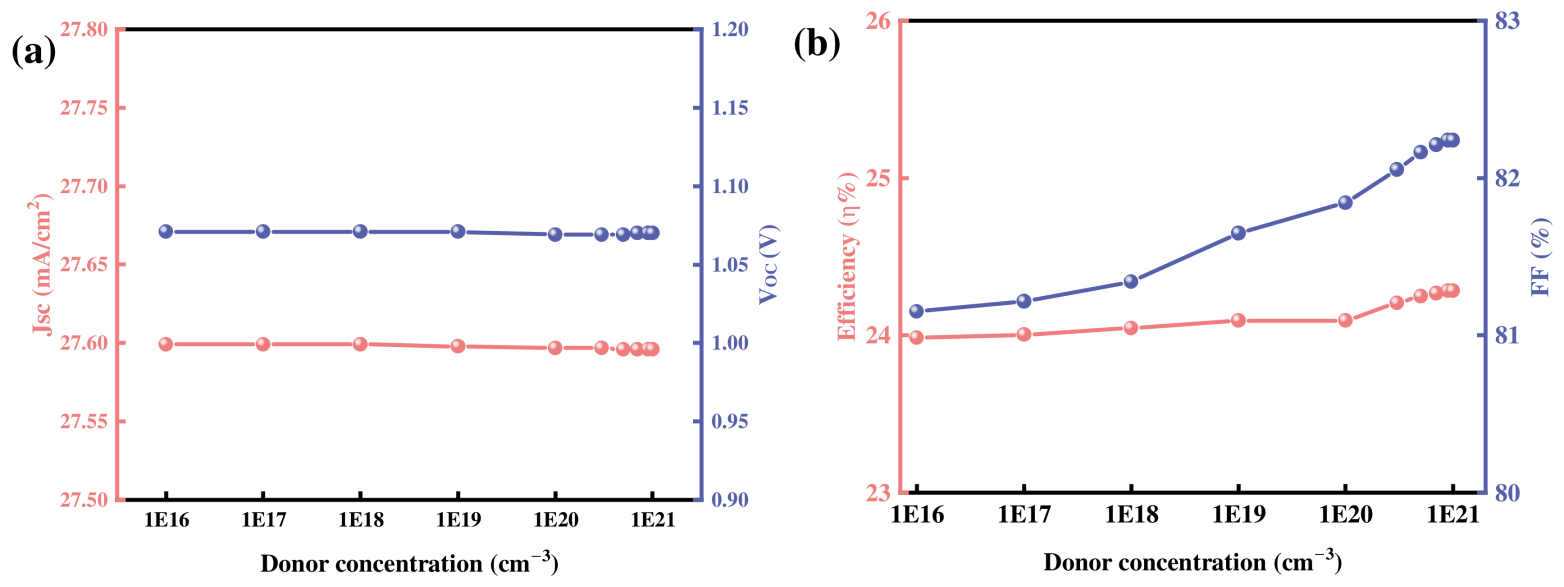
Effect of the donor concentration N_D_ of the Mg:TiO_2_ ETL on (**a**) V_OC_ and J_SC_ and (**b**) FF and PCE.

The donor concentration of the Mg:TiO_2_ ETL was varied from 10^16^ to 10^21^ cm^−3^ to evaluate its influence on device performance. The absorber thickness was fixed at the optimized value obtained above, while the other parameters were maintained unchanged. The resulting photovoltaic characteristics are presented in Figure 6. Over the investigated donor-concentration range, V_OC_ and J_SC_ remain nearly constant. V_OC_ varies only between approximately 1.069 and 1.071 V, while J_SC_ remains within 27.596–27.599 mA cm^−2^. This weak dependence is reasonable because variation in the ETL donor concentration does not directly alter the optical properties, thickness, or photogeneration characteristics of the MAPbI_3_ absorber.

In contrast, FF and PCE are more sensitive to N_D_. Increasing the donor concentration raises the majority-electron concentration in Mg:TiO_2_ and consequently enhances its electrical conductivity. Improved ETL conductivity can reduce resistive transport losses and facilitate electron collection at the front contact, which is mainly reflected in an increased FF. When N_D_ increases from 1 × 10^20^ to 9 × 10^20^ cm^−3^, the FF increases from 81.648% to 82.241%, while the PCE rises from 24.095% to 24.282%. Because V_OC_ and J_SC_ remain essentially unchanged, the improvement in PCE can primarily be attributed to the increase in FF [29]. Further increasing N_D_ to 10^21^ cm^−3^ produces almost no additional improvement, indicating that the benefit associated with increasing ETL conductivity has approached saturation under the present simulation conditions. Therefore, N_D_ = 9 × 10^20^ cm^−3^ was retained as a suitable donor concentration for the subsequent optimization. This analysis also confirms that the simulated performance of Mg:TiO_2_ is sensitive to the assumed donor concentration, although the improvement approaches saturation in the high-N_D_ regime. Electron mobility was not independently varied in the present study; therefore, the robustness of the Li/Co/Mg/Al ranking against simultaneous variations in N_D_ and mobility cannot be established and should be interpreted with caution.

#### 3.4.4. Effect of MAPbI_3_ Bulk Defect Density

**Figure 7:**
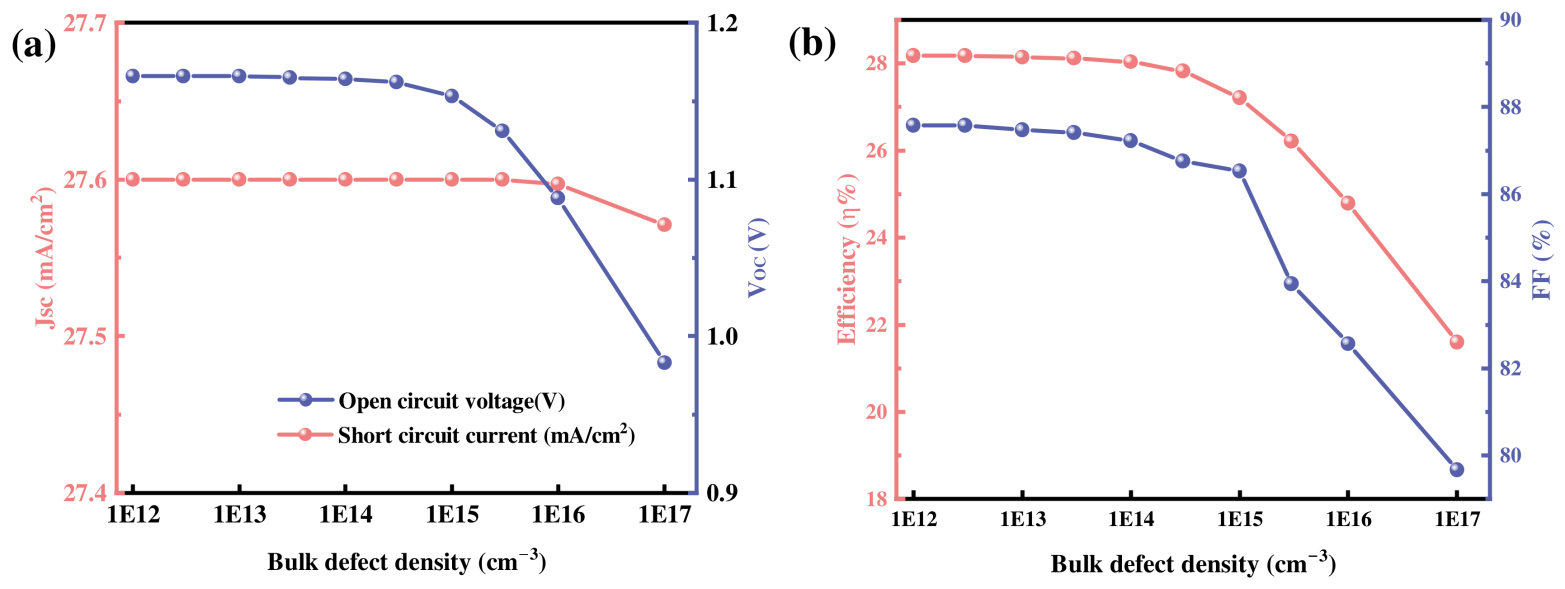
Effect of MAPbI_3_ bulk defect density N_t_ on (**a**) V_OC_ and J_SC_ and (**b**) FF and PCE.

Bulk defects in the perovskite absorber can introduce carrier-trapping and non-radiative recombination pathways and therefore strongly affect photovoltaic performance [39]. To evaluate this effect, the MAPbI_3_ bulk defect density N_t_ was varied from 1 × 10^12^ to 1 × 10^17^ cm^−3^ while the previously optimized device parameters were maintained. The calculated photovoltaic parameters are shown in Figure 7. At relatively low defect densities, the device performance is only weakly dependent on N_t_. Increasing N_t_ from 1 × 10^12^ to 1 × 10^13^ cm^−3^ decreases the PCE only slightly, from 28.179% to 28.140%. However, once N_t_ exceeds approximately 10^14^ cm^−3^, the degradation in device performance becomes increasingly pronounced. The PCE decreases from 28.033% at 1 × 10^14^ cm^−3^ to 24.785% at 1 × 10^16^ cm^−3^ and further to 21.595% at 1 × 10^17^ cm^−3^. At the highest defect density, V_OC_ and FF decrease to 0.983 V and 79.673%, respectively. In comparison, J_SC_ changes only slightly, from 27.600 to 27.571 mA cm^−2^ across the investigated range.

The strong reductions in V_OC_ and FF are consistent with enhanced defect-assisted recombination in the absorber. Within the defect formalism adopted in SCAPS-1D, increasing N_t_ increases the probability of carrier trapping and non-radiative recombination, thereby reducing carrier lifetime and quasi-Fermi-level splitting. The resulting increase in recombination losses lowers the achievable photovoltage and deteriorates the J-V characteristics. In contrast, the relatively small change in J_SC_ indicates that bulk defects predominantly affect carrier recombination and voltage-related losses rather than the initial photogeneration process. The sensitivity of PCE to N_t_ also reveals a saturation regime at low defect densities. Once N_t_ is reduced to approximately 10^13^ cm^−3^ or below, further reduction produces only a minor gain in efficiency. The highest simulated PCE of 28.179% is obtained at N_t_ = 1 × 10^12^ cm^−3^, with V_OC_ = 1.166 V, J_SC_ = 27.600 mA cm^−2^, and FF = 87.579%. This value was therefore adopted as the theoretical low-defect condition for the subsequent simulations.

It should be emphasized that N_t_ = 1 × 10^12^ cm^−3^ represents an idealized low-defect condition in the present model rather than a routinely achievable value for polycrystalline MAPbI_3_ films. Experimentally extracted defect densities remain strongly dependent on film quality and characterization method, with representative high-quality MAPbI_3_ films typically showing values above this level. Therefore, the performance obtained at N_t_ = 1 × 10^12^ cm^−3^ should be interpreted as a theoretical low-defect limit.

#### 3.4.5. Effect of Mg:TiO_2_/MAPbI_3_ Interface Defect Density

**Figure 8:**
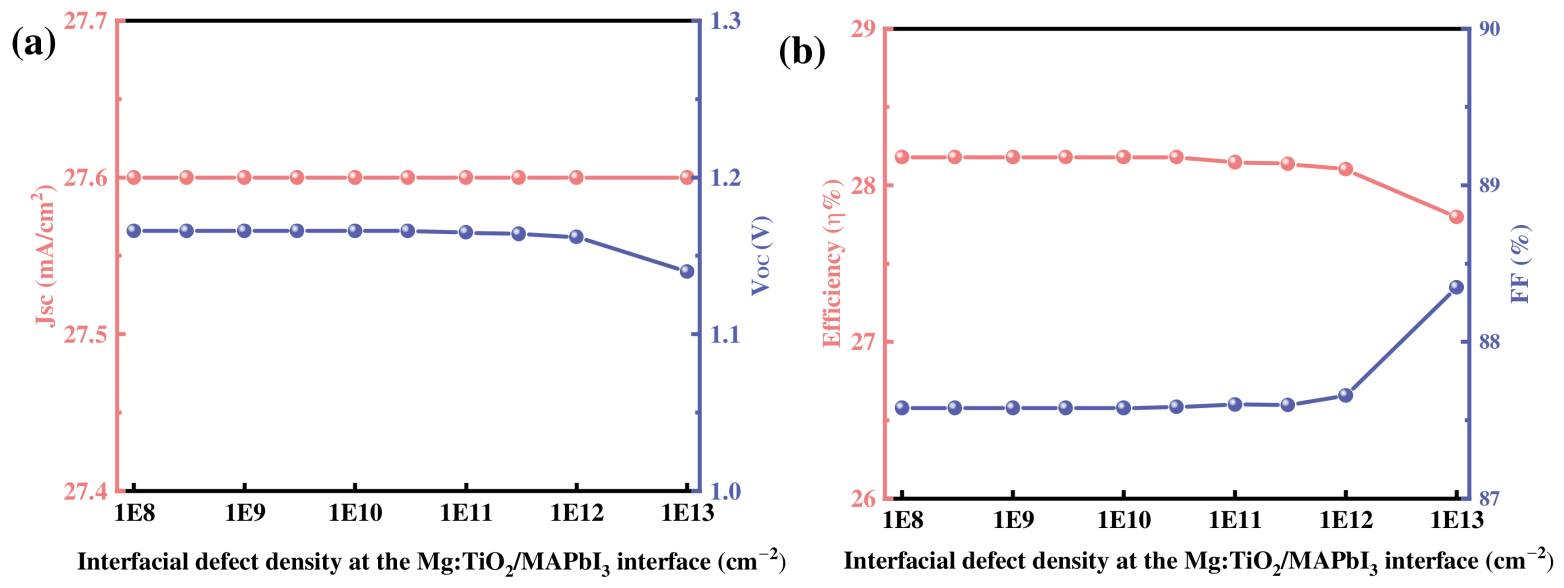
Effect of the Mg:TiO_2_/MAPbI_3_ interface defect density Nit on (**a**) V_OC_ and J_SC_ and (**b**) FF and PCE.

The defect density N_it_ at the Mg:TiO_2_/MAPbI_3_ interface was varied from 1 × 10^8^ to 1 × 10^13^ cm^−2^ to evaluate the sensitivity of the optimized device to interfacial defects. As shown in Figure 8, the photovoltaic parameters remain nearly unchanged when N_it_ is below approximately 10^10^ cm^−2^, with the PCE remaining close to 28.179%. When the interface defect density increases beyond approximately 10^11^ cm^−2^, the effect on V_OC_ becomes more apparent. V_OC_ decreases from approximately 1.166 V under low-defect conditions to 1.140 V at N_it_ = 10^13^ cm^−2^, while the PCE decreases from 28.179% to 27.796%. In contrast, J_SC_ remains essentially constant at approximately 27.600 mA cm^−2^. This behavior is consistent with the role of interface defect states as carrier-trapping and recombination centers. Increasing N_it_ enhances the probability of non-radiative recombination at the MAPbI_3_/Mg:TiO_2_ interface, reducing the quasi-Fermi-level splitting and consequently lowering V_OC_. The weak response of J_SC_ indicates that, within the investigated defect-density range and the interface parameters adopted in the model, the primary influence of interface defects is on recombination-related voltage losses rather than photogeneration.

Interestingly, FF does not decrease monotonically with N_it_ and shows a slight increase at the highest defect densities in the present simulation. Because this increase is insufficient to compensate for the loss in V_OC_, the overall PCE still decreases. Given that no independent analysis was performed to establish the physical origin of this FF variation, it is treated here as a model-specific response rather than evidence of beneficial high interface-defect densities. The device performance has essentially reached its low-defect plateau at N_it_ ≈ 10^10^ cm^−2^. Further reduction provides little additional improvement; therefore, 1 × 10^10^ cm^−2^ was selected as a representative optimized interface defect density for the subsequent simulation. In contrast to the very low bulk-defect condition, an interface defect density on the order of 10^10^ cm^−2^ is within the range inferred for well-controlled perovskite/TiO_2_ interfaces, although the exact value remains strongly dependent on interface preparation and the defect model used for extraction [40].

#### 3.4.6. Effect of Back-Contact Work Function

**Figure 9:**
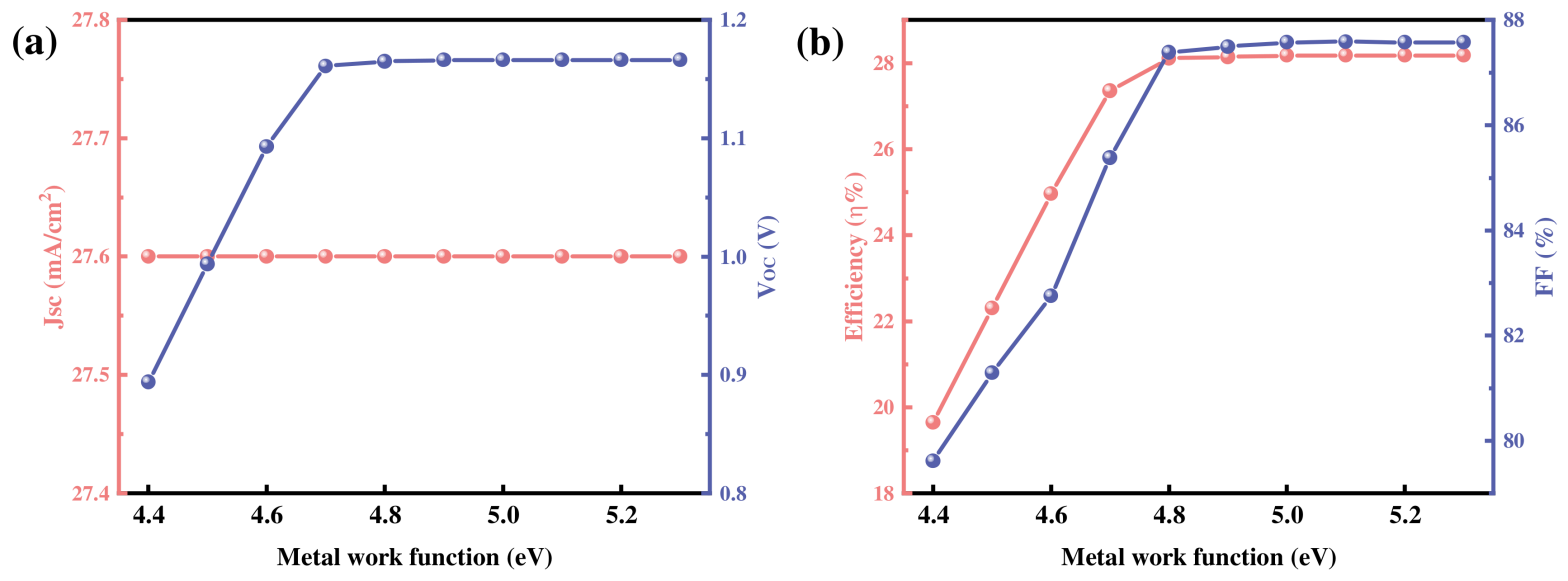
Effect of back-contact work function on (**a**) V_OC_ and J_SC_ and (**b**) FF and PCE.

The work function of the back contact strongly affects the energetic barrier for hole extraction from P_3_HT [41]. To investigate this effect, the back-contact work function was varied from 4.4 to 5.3 eV while the remaining optimized device parameters were kept unchanged. The corresponding photovoltaic responses are presented in Figure 9. Increasing the back-contact work function markedly improves V_OC_, FF, and PCE, whereas J_SC_ remains approximately constant at 27.600 mA cm^−2^. When the work function increases from 4.4 to 4.7 eV, V_OC_ increases from 0.894 to 1.161 V, FF from 79.619% to 85.387%, and PCE from 19.644% to 27.351%. This strong dependence originates from the energetic alignment between the p-type P_3_HT layer and the back electrode. At relatively low metal work functions, a substantial barrier to hole extraction is formed at the P_3_HT/metal contact, leading to less favorable carrier collection and increased contact-related losses. Increasing the metal work function reduces this energetic barrier and progressively promotes a near-ohmic contact for hole extraction.

According to the adopted P_3_HT parameters, its valence-band maximum can be estimated from E_V_ = −(χ + E_g_). With χ = 3.50 eV and E_g_ = 1.70 eV, E_V_ is approximately −5.20 eV. Accordingly, a back electrode with a work function approaching 5.1–5.2 eV provides more favorable energetic alignment with the P_3_HT valence band, facilitating hole collection at the back contact. The photovoltaic response gradually approaches saturation when the back-contact work function exceeds approximately 5.0 eV. At 5.0 eV, the device reaches a V_OC_ of 1.166 V, an FF of 87.579%, and a PCE of 28.179%. Further increasing the work function from 5.1 to 5.3 eV results in negligible additional changes, with the PCE remaining at approximately 28.18%. This saturation indicates that the hole-extraction barrier has been largely removed and that further increases in work function provide little additional benefit under the present simulation conditions. Considering both the simulated saturation behavior and the representative work function of Au, a value of 5.1 eV was adopted for the optimized back contact. Under the parameter combination obtained through this stepwise optimization procedure, the device achieves a V_OC_ of 1.166 V, a J_SC_ of 27.600 mA cm^−2^, an FF of 87.580%, and a PCE of 28.181%. This value represents the best performance obtained within the present sequential optimization procedure and should not be interpreted as a mathematically established global optimum.

### 3.5. Stepwise Performance Evolution

To make the origin of the efficiency gain more transparent, Table S6 summarizes the stepwise evolution of the photovoltaic parameters from the baseline TiO_2_/tetragonal-MAPbI_3_/P_3_HT device to the Mg:TiO_2_-based device and the subsequent sequential optimization stages. The comparison shows that the improvement from TiO_2_ to Mg:TiO_2_ is modest, whereas the largest increase in PCE is associated with the reduction in MAPbI_3_ bulk defect density. Representative experimental MAPbI_3_-based devices are also included for benchmarking. Because the experimental architectures and fabrication conditions are not fully identical to the present model, the comparison is intended to provide context rather than direct validation.

### 3.6. Comparison with Representative Experimental Devices

To place the simulated results in an experimental context, Table S7 compares the present SCAPS-1D results with representative experimentally reported MAPbI_3_ devices using TiO_2_/P_3_HT or Mg-modified TiO_2_ [29,35,42]. A directly identical Mg:TiO_2_/MAPbI_3_/P_3_HT architecture was not identified in the available literature; therefore, the closest experimentally relevant devices are included. The comparison is intended as a benchmark rather than a direct model validation because the experimental devices differ in transport-layer structure, fabrication route, and defect/interface quality.

## 4. Conclusions

A systematic SCAPS-1D simulation was conducted to investigate the effects of charge-transport-layer selection, MAPbI_3_ crystal phase, TiO_2_ modification, and key device parameters on the photovoltaic performance of MAPbI_3_-based perovskite solar cells. Among the 25 ETL/HTL combinations examined, TiO_2_/P_3_HT exhibited the best overall performance and was therefore selected as the reference transport-layer configuration. Comparison of cubic, orthorhombic, and tetragonal MAPbI_3_ showed that the tetragonal phase delivered the highest PCE of 23.653%, mainly because its higher J_SC_ compensated for its relatively lower V_OC_ and FF. Further evaluation of Li-, Co-, Mg-, and Al-doped TiO_2_ indicated that Mg:TiO_2_ provided the most pronounced and consistent improvement, increasing the PCE of the tetragonal device to 23.951%. The selected Glass/FTO/Mg:TiO_2_/tetragonal-MAPbI_3_/P_3_HT/Au device was subsequently optimized by varying the absorber thickness, Mg:TiO_2_ donor concentration, MAPbI_3_ bulk defect density, interface defect density, and back-contact work function. An absorber thickness of 0.911 μm provided a suitable balance between photocarrier generation and transport losses, while increasing the Mg:TiO_2_ donor concentration mainly improved the FF. The simulations further showed that device performance was particularly sensitive to the MAPbI_3_ bulk defect density and back-contact work function. Under the optimized conditions, the device reached a simulated V_OC_ of 1.166 V, J_SC_ of 27.600 mA cm^−2^, FF of 87.580%, and PCE of approximately 28.18%. Overall, these results demonstrate that coordinated optimization of absorber properties, electron-transport characteristics, defect density, and contact energetics can substantially improve the simulated performance of MAPbI_3_ perovskite solar cells. More importantly, the integrated analysis reveals that these factors affect different performance-limiting processes: the absorber phase mainly influences photocurrent generation, TiO_2_ modification primarily affects charge-transport losses, while defect density and contact energetics mainly govern recombination- and extraction-related losses. This distinction provides a more physically informed basis for prioritizing device optimization and narrowing the experimental design space.

## Figures and Tables

**Table 1:** Performance of FTO/TiO_2_/MAPbI_3_ (three crystal phases)/P_3_HT devices.

Device	V_OC_ (V)	J_SC_ (mA/cm^2^)	FF (%)	PCE (%)
MAPbI_3_ (cubic)	1.153	20.221	83.557	19.484
MAPbI_3_ (orthorhombic)	1.147	22.473	83.778	21.599
MAPbI_3_ (tetragonal)	1.089	26.343	82.465	23.653

**Table 2:** Photovoltaic parameters of MAPbI_3_ devices employing undoped and metal-doped TiO_2_ ETLs.

Device	Voc (V)	J_SC_ (mA/cm^2^)	FF (%)	PCE (%)
Li: TiO_2_/MAPbI_3_(cubic)/P_3_HT	1.152	20.199	84.591	19.682
Li: TiO_2_/MAPbI_3_(orthorhombic)/P_3_HT	1.146	22.450	84.594	21.762
Li: TiO_2_/MAPbI_3_(tetragonal)/P_3_HT	1.087	26.319	83.092	23.768
Co: TiO_2_/MAPbI_3_(cubic)/P_3_HT	1.152	20.200	84.556	19.675
Co: TiO_2_/MAPbI_3_(orthorhombic)/P_3_HT	1.146	22.451	84.561	21.755
Co: TiO_2_/MAPbI_3_(tetragonal)/P_3_HT	1.087	26.321	83.055	23.760
Mg: TiO_2_/MAPbI_3_(cubic)/P_3_HT	1.153	20.220	85.103	19.834
Mg: TiO_2_/MAPbI_3_(orthorhombic)/P_3_HT	1.147	22.472	85.058	21.919
Mg: TiO_2_/MAPbI_3_(tetragonal)/P_3_HT	1.087	26.341	83.612	23.951
Al: TiO_2_/MAPbI_3_(cubic)/P_3_HT	1.153	20.221	83.583	19.490
Al: TiO_2_/MAPbI_3_(orthorhombic)/P_3_HT	1.147	22.473	83.805	21.607
Al: TiO_2_/ MAPbI_3_(tetragonal)/P_3_HT	1.089	26.343	82.491	23.660

## Data Availability

Data are contained within the article.

## Supplementary Materials

The following supporting information can be downloaded at: https://www.mdpi.com/article/10.3390/nano16191237/s1; Table S1. Material parameters for HTLs; Table S2. Material parameters for ETLs; Table S3. Interfacial defect parameters for MAPbI_3_-based perovskite solar cells; Table S4. Material parameters for the three crystal phases of MAPbI_3_ and FTO; Table S5. Material parameters of TiO_2_ with different metal dopants; Table S6. Stepwise evolution of simulated photovoltaic performance and comparison with representative experimental MAPbI_3_-based devices; Table S7. Comparison of the present simulated device performance with representative experimental MAPbI_3_ perovskite solar cells; Figure S1. Schematic diagram of the Glass/FTO/TiO_2_/MAPbI_3_/P_3_HT/Au perovskite solar cell structure.
