# Supplementary material for: Numerical Simulation of the Effect of Mg-Doped TiO2 Electron Transport Layer on the Performance of Tetragonal MAPbI3 Perovskite Solar Cells
Source: Nanomaterials (Basel). 2026 Sep 30;16(19):1237. doi: 10.3390/nano16191237 (PMC13667230; doi:10.3390/nano16191237)
Supplement: Supplementary file 1 [file nanomaterials-16-01237-s001.zip › nanomaterials-4575011-supplementary.pdf]

Numerical Simulation of the Effect of Mg-Doped TiO_2 Electron Transport Layer on the Performance of Tetragonal $MAPbI_3$ Perovskite Solar Cells

Chunlei Shi [1], Xuan Yu [1], Ji Liu [2], Zhiyuan Chen [1] and Jian Jiao [1,*]

[1] College of Physics, Changchun Normal University, Changchun 130032, China
[2] Changchun Jilin University Zhengyuan Information Technologies Co., Ltd., Changchun 130012, China
* Correspondence: jiaojian@ccsfu.edu.cn

Table S1. Material parameters for HTL layers.

Parameters	P_3HT	Zn_3P_2	CBTS	$CuSbS_2$	GO
Thickness (μm)	0.02	0.02	0.02	0.02	0.02
Band gap energy (eV)	1.70	1.50	1.90	1.58	2.48
Electron affinity (eV)	3.50	4.20	3.60	4.20	2.30
Dielectric Permittivity	3.00	7.11	5.40	14.60	10.00
(Conduction Band) effective density of states (cm^{-3})	2.00E+21	2.20E+18	2.20E+18	2.00E+18	2.20E+18
(Valence Band) effective density of states (cm^{-3})	2.00E+211	1.80E+19	1.80E+19	1.00E+18	1.80E+19
Electron thermal velocity (cm/s)	1.00E+7	1.00E+7	1.00E+7	1.00E+7	5.00E+7
Hole thermal velocity (cm/s)	1.00E+7	1.00E+7	1.000E+7	1.00E+7	5.00E+7
Electron mobility (cm^2/Vs)	1.86E-2	1.00E+0	3.00E+1	4.90E+1	2.60E+1
Hole mobility (cm^2/Vs)	1.80E-6	1.00E+1	1.00E+1	4.90E+1	1.23E+2
Shallow uniform donor density N_D (cm^{-3})	0.00E+0	0.00E+0	0.00E+0	0.00E+0	0.00E+0
Shallow uniform acceptor density N_A (cm^{-3})	1.00E+18	1.00E+19	1.00E+15	1.00E+18	2.00E+18
Total density (cm^{-3})	1.00E+15	1.00E+14	1.00E+14	1.00E+15	1.00E+9

Table S2. Material parameters for ETL layers.

Parameters	TiO_2	SnS_2	WS_2	C_{60}	MoO_3
Thickness (μm)	0.02	0.02	0.02	0.02	0.02
Band gap energy (eV)	3.20	2.24	1.80	1.70	3.00
Electron affinity (eV)	4.00	4.24	3.95	3.90	2.30
Dielectric Permittivity	9.00	10.00	13.60	4.20	18.00
(Conduction Band) effective density of states (cm^{-3})	2.00E+18	2.20E+18	1.50E+18	8.00E+19	1.00E+19
(Valence Band) effective density of states (cm^{-3})	1.80E+19	1.80E+19	1.80E+19	8.00E+19	2.20E+18
Electron thermal velocity (cm/s)	1.00E+7	1.00E+7	1.00E+7	1.00E+7	1.00E+7
Hole thermal velocity (cm/s)	1.00E+7	1.00E+7	1.00E+7	1.00E+7	1.00E+7
Electron mobility (cm^2/Vs)	2.00E+1	5.00E+1	1.00E+2	8.00E-2	2.10E+2
Hole mobility (cm^2/Vs)	1.00E+1	5.00E+1	1.00E+2	3.50Ecm-3	2.10E+2
Shallow uniform donor density N_D (cm^{-3})	9.00E+16	1.00E+17	1.00E+18	1.00E+17	1.00E+18
Shallow uniform acceptor density N_A (cm^{-3})	0.00E+0	0.00E+0	0.00E+0	0.00E+0	0.00E+0
Total density (cm^{-3})	1.00E+15	1.00E+14	1.00E+14	1.00E+15	1.00E+15

Table S3. Interfacial defect parameters for $MAPbI_3$-based perovskite solar cells.

Parameters	$MAPbI_3$/HTL	ETL/$MAPbI_3$
Defect type	Neutral	Neutral
Capture cross section for the electron (cm^2)	1.00E-19	1.00E-19
Capture cross section for a hole (cm^2)	1.00E-19	1.00E-19
Energy distribution	Single	Single
Reference for defect energy level Et	Above the highest EV	Above the highest EV
Energy with respect to Reference (eV)	0.60	0.60
Character energy (eV)	0.10	0.10
Total density (cm^{-2})	1.00E+10	1.00E+10

Table S4. Material parameters for the three crystal phases of MAPbI3, and FTO.

Parameters	FTO	$MAPbI_3$ (cubic)	$MAPbI_3$ (orthorhombic)	$MAPbI_3$ (tetragonal)
Thickness (μm)	0.02	0.60	0.60	0.60
Band gap energy (eV)	3.50	1.70	1.62	1.50
Electron affinity (eV)	4.00	3.90	3.90	3.90
Dielectric Permittivity	9.00	10.00	8.0	8.50
(Conduction Band) effective density of states (cm^{-3})	2.20E+18	2.76E+18	2.76E+18	2.76E+18
(Valence Band) effective density of states ($1/cm^3$)	1.80E+19	3.90E+19	3.90E+19	3.90E+19
Electron thermal velocity (cm/s)	1.00E+7	1.00E+7	1.00E+7	1.00E+7
Hole thermal velocity (cm/s)	1.00E+7	1.00E+7	1.00E+7	1.00E+7
Electron mobility (cm^2/Vs)	2.00E+2	1.50E+1	1.50E+1	1.50E+1
Hole mobility (cm^2/Vs)	1.00E+2	1.50E+1	1.50E+1	1.50E+1
Shallow uniform donor density N_D (cm^{-3})	1.00E+19	0.00E+0	0.00E+0	0.00E+0
Shallow uniform acceptor density N_A (cm^{-3})	0.00E+0	1.000E+11	1.00E+11	1.00E+11
Total density (cm^{-3})	1.00E+15	1.50E+16	1.50E+16	1.50E+16

Table S5. Material parameters of TiO_2 with different metal dopants.

Parameters	Co: TiO_2	Al: TiO_2	Li: TiO_2	Mg: TiO_2
Thickness (μm)	0.02	0.02	0.02	0.02
Band gap energy (eV)	3.40	3.12	3.40	3.22
Electron affinity (eV)	3.90	4.20	3.90	4.20
Dielectric Permittivity	12.00	13.60	13.60	13.60
(Conduction Band) effective density of states (cm^{-3})	3.00E+20	2.20E+18	3.00E+20	2.20E+18
(Valence Band) effective density of states (cm^{-3})	2.00E+20	2.20E+18	2.00E+20	2.20E+18
Electron thermal velocity (cm/s)	1.00E+7	1.00E+7	1.00E+7	1.00E+7
Hole thermal velocity (cm/s)	1.00E+7	1.00E+7	1.00E+7	1.00E+7
Electron mobility (cm^2/Vs)	3.30E+0	2.00E+1	2.00E-2	2.00E+1
Hole mobility (cm^2/Vs)	1.00E-3	1.00E+1	1.00E-3	1.00E+1
Shallow uniform donor density N_D (cm^{-3})	1.00E+17	1.000E+17	1.00E+17	9.00E+20
Shallow uniform acceptor density N_A (cm^{-3})	0.00E+0	0.00E+0	0.00E+0	0.00E+0
Total density (cm^{-3})	1.10E+15	1.00E+15	2.30E+15	1.00E+10

Table S6. Stepwise evolution of simulated photovoltaic performance and comparison with representative experimental $MAPbI_3$-based devices.

Optimization stage	V_{OC} (V)	ΔV_{OC} (V)	J_{SC} (mA/cm^2)	ΔJ_{SC} (mA/cm^2)	FF (%)	ΔFF (%)	PCE (%)	ΔPCE (%)
Baseline: Glass/FTO/TiO_2/$MAPbI_3$ (tetragonal)/P_3HT/Au	1.089	–	26.343	–	82.465	–	23.653	–
Mg:TiO_2 replacing TiO_2	1.087	-0.002	26.341	-0.002	83.612	+1.147	23.951	+0.298
$MAPbI_3$ absorber thickness optimization	1.070	-0.019	27.594	+1.251	82.241	-0.224	24.282	+0.331
Mg:TiO_2 donor concentration optimization	1.070	-0.019	27.594	+1.251	82.241	-0.224	24.282	0.000
$MAPbI_3$ bulk defect density optimization	1.166	+0.077	27.600	+1.257	87.580	+5.115	28.179	+0.387
$MAPbI_3$/Mg:TiO_2 interface defect density optimization	1.166	+0.077	27.600	+1.257	87.580	+5.115	28.179	0.000
Back-contact work function optimization	1.166	+0.077	27.600	+1.257	87.580	+5.115	28.181	+0.002
Final optimized	1.166	+0.077	27.600	+1.257	87.580	+5.115	28.181	+4.528

Table S7. Comparison of the present simulated device performance with representative experimental $MAPbI_3$ perovskite solar cells.

Device	Type	V_{OC} (V)	J_{SC} (mA/cm^2)	FF (%)	PCE (%)	Ref.
FTO/$MgTiO_3$@TiO_2/$MAPbI_3$/Carbon	Experiment	0.95	22.62	48.00	10.39	[42]
FTO/TiO_2/$MAPbI_3$/Carbon	Experiment	0.80	14.05	44.20	4.98	[42]
FTO/Al-TiO_2/$MAPbI_3$/Spiro-OMeTAD/Ni	Simulation	1.346	18.220	78.500	19.260	[29]
FTO/Mg-TiO_2/$MAPbI_3$/Spiro-OMeTAD/Ni	Simulation	1.170	23.174	72.740	19.860	[29]
FTO/TiO_2/Perovskite/Spiro-OMeTAD/Au	Experiment	1.06	21.36	72.30	17.29	[35]
FTO/Mg-TiO_2/Perovskite/Spiro-OMeTAD/Au	Experiment	1.101	22.56	76.80	19.80	[35]
TiO_2/$MAPbI_2$/P_3HT	Simulation	1.089	26.343	82.465	23.653	This work
Mg:TiO_2/$MAPbI_2$/P_3HT	Simulation	1.087	26.341	83.612	23.951	This work

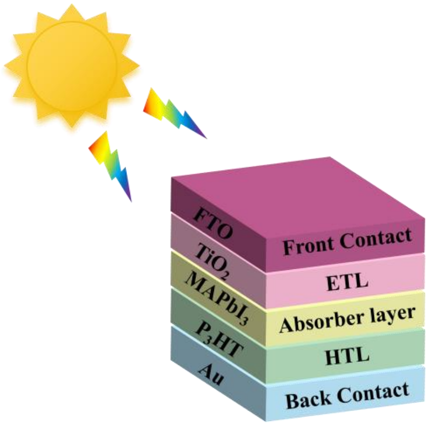

Figure S1. Schematic diagram of the Glass/FTO/TiO_2/$MAPbI_3$/P3HT/Au perovskite solar cell structure.
